# Supplementary material for: Are carbon emissions trading and green financial instruments synergistic? -Comprehensive quantitative research based on content analysis
Source: PLoS One. 2024 Mar 7;19(3):e0298601. doi: 10.1371/journal.pone.0298601 (PMC10919601; doi:10.1371/journal.pone.0298601)
Supplement: S1 Data — (ZIP) [file pone.0298601.s001.zip › Data/地级市Z_F工作报告.zip!/░▓╗╒/╗┤─╧╩╨/└┤╫╘╣1⁄2╓┌║┼í╛┬φ┐╦╩2╛▌═°í┐_2010.docx]

各位代表：

      现在，我代表市人民政府向大会报告工作，请予审议，并请市政协委员和其他列席人员提出意见。

**一、化危为机，负重奋进的2009年**

      2009年是极不平凡的一年。我市经济社会发展经受住了严峻挑战和重大考验。全市人民在市委的坚强领导下，深入学习实践科学发展观，迎难而上，奋力拼搏，危中寻机，化危为机，实现了经济社会平稳较快发展，圆满完成了市十四届人大三次会议确定的年度目标任务。

      预计，全市生产总值突破500亿元，增长13%以上；财政收入75亿元，增长24.7%；固定资产投资300亿元，增长33%；社会消费品零售总额159亿元，增长18.7%；城镇居民人均可支配收入14000元，增长7.7%；农民人均纯收入5000元，增长12.6%。新增城镇就业岗位3.7万个，城镇登记失业率4.43%；人口自然增长率6.8‰。主要经济指标提前一年实现“十一五”目标。

      一年来，我们紧紧围绕保增长、保民生、保稳定，主要做了以下工作：

**（一）抓工业、保企业，政策效应加速显现**

      去年以来，面对国际金融危机和资源型城市可持续发展的双重压力，针对年初工业生产下滑、投资增幅下降、企业效益走低的严峻形势，我们果断决策、及时应对，相继出台了促进经济平稳较快发展、非公经济、财税扶持、自主创新等9个方面共400余条政策措施，安排财政扶持资金1.5亿元，实施结构性减税6亿元。取消行政事业收费114项、降低收费标准15项，减收3860万元。阶段性调整了社会保险缴费政策，减收3632万元，缓缴8484万元。建立规模以上工业企业及项目市级领导联系人制度，实施“224”帮扶工程。这些措施对缓解经济运行中的突出矛盾、保持经济平稳较快发展，发挥了至关重要的作用。

     工业保持较快增长。预计规模以上工业企业完成增加值280亿元，增长19%。编制了七大产业调整振兴规划，制定了工业企业“退城进园”三年计划。全年新增规模以上工业企业102户，总数达350户。张集西风井、新庄孜煤矸石电厂等一批重点项目建成投产。预计原煤产量7960万吨，发电量440亿千瓦时。煤化工基地建设取得重大进展。煤化工新区一期12.7平方公里的基础设施建设、拆迁安置等工作全面推进。与中石化签订了合作协议，首期计划投资200亿元，为煤化工发展和产业结构调整提供了强力支撑。淮化清洁生产工艺项目竣工。启动了淮南高新技术产业开发区规划建设，起步区1.8平方公里路网、国家煤及煤化工产品质检中心、科技创新公共服务中心开工建设。3个省级开发区扩容规划和4个县区工业集聚区规划编制完成。比塞洛斯煤机、长壁煤机、汇通纺织等一批重点项目开工。淮南农场城乡一体化综合改革试验区和淮南垦区建设投资公司挂牌成立。县区工业集聚区加快建设，毛集省级民营科技产业园揭牌。

      节能减排成效显著。荣获“全国节能减排二十佳城市”。加快实施热电联产、建筑节能、绿色照明等十大重点节能工程。东部垃圾处理场投入使用，16个污染减排项目、7个村镇生态式污水处理站全面建成，市医疗废物处置中心奠基。单位生产总值能耗下降5%，化学需氧量削减6%，二氧化硫排放量低于控制指标2.8个百分点，连续三年超额完成省下达的目标任务。获得安徽省淮河巢湖流域水污染防治年度考核第一名。

**（二）抓投资、促消费，发展空间加速拓展**

      突出把投资作为扩内需的重要抓手。坚持项目、投资、招商“三位一体”突破，以项目带动投资。成立了高规格的市项目谋划与推进工作委员会，安排项目编制补助和激励资金1500万元。完善了“9771”项目库，储备项目957个，投资规模2700亿元；建立了工业、城建、农业、服务业项目库，储备项目629个，投资规模1432亿元。全年实施项目960个，其中新开工项目740个、省“861”项目114个。实施千万元以上技改项目43个。争取中央新增投资7.08亿元，261个项目获得支持。

      突出把融资作为扩内需的重要途径。出台了金融支持经济发展、中小企业信贷支持等政策措施，发放财政贴息资金1400万元。预计全年各金融机构新增贷款133.8亿元，是上年的5倍。组建了淮南通商农村合作银行，引进了浦发银行、招商银行，潘集农村信用联社改制工作顺利推进。获批小额贷款公司11家，其中开业2家。培育担保机构50家，其中亿元以上4家。大力开展银企对接活动，对接资金517.5亿元。城投、产投、山南投资公司融资工作实现历史性突破，获银行授信200亿元，融资52.4亿元，利用外国政府贷款5900万美元，���城市建设和产业发展提供了有力支撑。

      突出把消费作为扩内需的重要动力。制定了扩大内需促进消费的实施意见。世贸商城等项目开工建设，家乐福商务广场建成开业。家电、汽车摩托车下乡活动深入开展，发放财政补贴2141万元。社区商业“双进”工程、“万村千乡”市场工程、“新网工程”扎实推进，新增农家店180个、“千村百镇”示范村44个。“早餐工程”供应网络基本建成。文化旅游消费不断升温，实现旅游收入20亿元，增长20%。

**（三）抓规划、大建设，城市品位加速提升**

      坚持规划先行。完成了新一轮城市总体规划和土地利用总体规划修编。完成了老城区道路网改善和城市主要道路夜景照明、建筑特色与色彩、文化设施、环境卫生等一批专项规划，编制完成了8个控制性详细规划，规划体系不断完善，规划质量实现了历史性突破。

      启动了城市大建设。全年实施项目220个，完成投资150亿元，是前5年的总和。制定并实施山南新区开发建设及老城区市政基础设施提升三年计划、城市大建设三年投融资计划。山南新区加快建设。国投新集总部竣工投用，市级机关办公楼、武警新营区等进展顺利，南纬二路一期等21条道路加快建设，奥林匹克公园、山南污水处理厂、淮南二中、山南印象、人民公园、政务服务中心等一批项目开工。老城提升加快推进。洞山中路、陈洞路、国庆东路、蔡新路等20条道路改扩建工程建成通车，淮舜南路、夏郢西路二期、林场路等一批道路开工建设，老城区路网加密40个项目完成设计招投标。23个旧改项目加快推进。合淮蚌铁路客运专线、淮蚌高速公路、东西部第二通道、206国道改线、孔李淮河大桥引桥及引线开工建设，淮河二桥、淮上大桥、凤台二桥、淮南大道、九龙大道等78个项目前期工作加快推进。龙湖公园改造一期、八公山地质展示馆、八公山豆腐文化博物馆、新四军纪念林陈列馆等建成开放，焦岗湖滨湖度假村等21个项目开工建设。房地产新开工面积200万平方米，竣工120万平方米。市体育文化中心比赛馆工程荣获“鲁班奖”。与山东志高集团签订了建设文化科技动漫产业园合作协议。县区大建设全面启动。全年报批建设用地12231亩，整理土地9941亩，置换土地7140亩，清理闲置土地2547亩。大建设成为经济寒潮中的“报春花”和支撑发展的“新引擎”。

      城市创建取得新成果。荣获中国优秀旅游城市称号。新增焦岗湖、上窑2个国家4A级旅游景区，焦岗湖湿地被批准建设国家湿地公园。积极推行网格化管理，建成东部城区数字化城管系统。深入开展“净化社区、美化街巷”活动，洞山中路、陈洞路等绿化亮化一体化改造全面完成。启动了全国绿化模范城市创建工作。新增城市绿地面积80.6万平方米，完成植树造林2.34万亩，建成合淮阜高速公路淮南段森林生态长廊。凤台八一林牧场被命名为全省“森林旅游人家”，获得全国表彰。

**（四）抓“三农”、强基础，城乡统筹加速推进**

      现代农业稳步发展。出台了促进现代农业发展的若干意见。全市财政用于“三农”支出14亿元，增长20.7%。奋力夺取抗旱保苗的全面胜利，全年粮食总产137万吨，增长2.2%，创历史新高。肉蛋奶总产17.9万吨，增长10%。水产品产量6.5万吨，增长9%。蔬菜产量65.9万吨，增长5.8%。农业产业化“712”提升行动计划深入实施，全市农产品加工产值81亿元，增长35%。新增国家级绿色食品原料基地1个、省级农产品标准化示范区2个、规模养殖场18个。新增无公害农产品、绿色食品、有机食品26个。升级改造农贸市场2个。新增农民专业合作社172家，2家被评为省先进合作社。在全省率先开通了“农业114”网络销售平台。

      新农村建设扎实推进。建成农村公路392.5公里，提前完成“十一五”村村通计划。新建客运站和招呼站262个，改造渡口22个。新建供水工程53处，解决11万人安全饮水问题。建成沼气池4.2万立方米。治淮新三项工程、大型泵站技改等项目扎实推进，石姚段、洛河洼行洪堤退建加固主体工程完成90%。完成6座水库除险加固工程，完成泥河泵站、禹王泵站上划市管工作。坚持城乡环保一体化，深入实施“清洁乡村、美化家园”示范工程，51个示范村村容村貌大幅改观。创建省级环境优美乡镇7个、省级生态示范村10个。1县4区成立土地流转服务中心，流转土地8.5万亩。我市被批准为全省城乡一体化综合配套改革试点市。

**（五）抓开放、促改革，发展活力加速集聚**

      深化合作取得新突破。合淮同城化总体规划通过专家评审，合淮工业走廊规划、合肥经济圈城镇体系规划编制完成，交通、旅游、信息等对接全面展开。积极开展与六安、巢湖的合作。创造性地融入皖江城市带承接产业转移示范区。与央企合作成果丰硕，对接签约项目25个，投资规模1466亿元。淮南矿业集团参股省内外16家电厂，电力权益规模达1060万千瓦。

      对外开放呈现新局面。荣获“2009浙商投资（中国）最佳城市”、“2009中国吸引华商投资最具潜力城市”称号。制定了统筹“五外”工作、加快对外开放的若干意见，出台了进一步加快外经贸发展的实施意见。在全省率先成立了市外商投资服务局，组建了251个招商小分队，积极推行县区“一把手”招商。组团参加了中博会、高交会等重要节会，赴香港、深圳等地开展大规模招商推介活动。淮南福建商会成立。成功举办第二届中国淮南国际少儿艺术节、第十六届中国豆腐文化节。先后引进了中石化、中国城建、中国太平洋、郑州煤机、大连正源、海螺集团等一批大企业大集团。全年引进内资165亿元，利用外资1.2亿美元，完成进出口总额1.2亿美元。

      各项改革取得新进展。3户企业完成股改，进入券商辅导阶段。不良金融资产打包处置工作扎实开展。市政府机构改革基本完成。在全省率先完成工商系统脱钩改革任务。9个镇被列入省“扩权强镇”试点，45个乡镇实行分类考核。启动村级公益事业一事一议、农村公路管理养护体制改革。凤台县被列为全省农村土地使用综合改革试点县。出台了扶持西部城区、毛集实验区和山南七乡镇改革发展的政策措施。

      自主创新取得新成效。第六次荣获全国科技进步先进市。科技经费支出增长25.6%。“四点一园”自主创新高地加快建设。落户淮化的流化床甲醇制丙烯（FMTP）工业技术处于国际领先水平。鉴定科技成果42项，获得国家科技进步奖2项、省科技进步奖7项，专利申请797件，专利授权271件，认定高新技术企业10家、高新技术产品24个、省创新型试点企业3家，成功申报国家创新基金项目14个。立项建设4个省级工程技术研究中心。积极构建共享共用的大型科学仪器设备平台。加强质量兴市工作，设立“市长质量奖”，新增省名牌产品5个。深入实施人才强市战略。淮南矿业集团袁亮同志当选中国工程院院士。

**（六）抓民生、促和谐，人民生活加速改善**

      民生工程扎实推进。坚持把民计民生作为政府工作的出发点和落脚点，强化组织领导，落实资金保障，狠抓督查调度，创新推进机制，全面完成了35项民生工程年度目标任务，累计投入资金7.7亿元，增长63.8%，惠及全市220万人，推动了社会建设，促进了社会和谐。

      就业和社会保障工作进一步加强。出台了进一步做好就业工作的实施意见，4个省级农民工创业园加快建设。实现下岗失业人员再就业2.01万人、农村劳动力转移就业3.01万人。完成各类职业培训4万人。劳动合同签订率97.1%。实施社会救助150.4万人次，发放救助金1.77亿元。新建、改扩建农村敬老院18所，农村五保集中供养率达30％。被征地农民养老保险实现市级统筹，凤台县纳入国家新型农村社会养老保险试点。完成保障性住房投资12.6亿元，建设经济适用房28.25万平方米、廉租住房9.78万平方米，棚户区改造190万平方米，发放廉租住房补贴4822户共1083万元。

      采煤沉陷区综合治理扎实推进。成立市采煤沉陷区综合治理办公室，并将其作为政府工作部门。建立了采煤沉陷区环境综合治理机制，在全省率先出台了采煤沉陷区农村集体土地居民补偿搬迁安置暂行办法。投入10.65亿元，开工建设搬迁安置点19个，搬迁居民11854人。安排6000万元，实施采煤沉陷区农民培训就业援助行动。

      促进教育公平取得新进展。提高了义务教育公用经费标准。加快中小学布局调整，撤并学校31所。创建标准化学校100所，全面启动校舍安全工程。建成市青少年校外教育活动中心。职业教育加快发展，高中阶段教育毛入学率达76%，比上年提高6.6个百分点。

      卫生服务体系不断完善。医药卫生体制改革启动。新建和改扩建城乡卫生机构139个。城镇职工、城镇居民参加基本医疗保险人数分别达42.6万人、51.9万人，城镇居民基本医疗保险实现市级统筹。新型农村合作医疗参合率95.28%，大通、田家庵、谢家集、八公山四区实现市级统筹。抓好重大疾病防治和应急处置工作，有效防控甲型H1N1流感。食品药品监管不断强化。

      文化体育等事业加快发展。《淮南市非物质文化遗产丛书》出版，4人被确定为国家级非物质文化遗产传承人。建成农家书屋93个，发放文化信息资源共享工程设备798套。建成农民体育健身工程39个。成功举办了市第六届运动会。广播电视事业不断发展，对外宣传全面提升。深入开展拥军优属、军民共建活动，荣获全省双拥模范城七连冠。民族工作扎实有效，荣获全国民族团结进步模范集体称号。妇女儿童、老年人、残疾人事业得到新发展，统计、档案、地方志、防震减灾取得新进步，外事、侨务、对台工作呈现新局面，国家安全、国防动员、人民防空、民兵预备役建设迈上新台阶。

      维护社会稳定的各项工作得到加强。扎实开展“维稳综治基层基础夯实年”活动，深入推进基层平安创建。始终保持对刑事犯罪活动的高压态势，组织开展打黑除恶、命案侦破等专项斗争，全市社会治安形势持续好转。扎实做好矛盾纠纷排查调处和信访稳定工作，把不稳定因素消除在萌芽状态。全面落实“安全生产年”总体部署，扎实推进“三项行动”，小煤矿实行分类管理，安全生产形势总体平稳。应急管理体制机制不断完善，预防和处突能力不断提高。

**（七）抓服务、优环境，工作作风加速转变**

      坚持依法行政，推进管理创新，加强效能建设，进一步提升服务发展、服务基层、服务群众的能力和水平。全面开展问计于民活动。强力推进“两集中、两到位”改革，开展“只进一个门、只找一个人”活动，29个部门的行政审批科室完成整合，全部进驻政务中心，审批科室由115个精简到29个，审批时限平均压缩4.9个工作日。基层民主不断扩大，202个社区完成换届选举，其中14个社区实行直选。自觉接受人大法律监督和政协民主监督，建立了目标管理、政务监督、行政监察、审计监督和舆论监督“五位一体”的大督查机制。办理市人大代表议案、建议129件，市政协委员提案418件。制定政府规章3件，提请市人大常委会审议地方性法规3件。认真开展加强行政决策机制建设示范市工作。制定了加强新形势下政府自身建设的若干意见。完善“政务信息公开系统”，开通网上协同办公系统。对市政府公务用车实行GPS管理。全面落实党风廉政建设责任制，反腐倡廉工作力度不断加大，实现了经济社会发展与党风廉政建设的“双促进、双提升”。

      各位代表，过去一年，取得的成绩来之不易。这是省委、省政府和市委正确领导的结果，是市人大和市政协关心支持的结果，是全市人民奋发进取的结果。在此，我代表市人民政府，向全市人民及驻淮部队、武警官兵、公安民警，向各民主党派、工商联、无党派和各界人士，向关心支持淮南现代化建设的驻淮单位及海内外朋友，表示衷心地感谢！

      在充分肯定成绩的同时，我们也清醒地看到经济社会发展中的矛盾和问题：一是受金融危机的冲击和影响，工业企稳回升的基础还不牢固，部分行业产值和效益下降，扭转外资外贸下滑的任务相当艰巨。二是产业结构不合理，非煤产业比重较低，服务业、高新技术产业等发展不充分，自主创新能力不强。三是县区域经济和非公经济发展不足，园区经济发展滞后。四是统筹城乡发展压力加大，采煤沉陷区综合治理、农村公共服务建设、资源节约和环境保护难度增加。五是安全生产形势不容乐观，信访稳定压力加大，发展环境仍需优化。六是政府自身建设有待进一步加强。对此，我们务必高度重视，认真加以解决。

**二、团结拼搏，扎实做好2010年各项工作**

      2010年是巩固扩大经济企稳回升势头、全面完成“十一五”规划任务的关键之年，也是奋力赶超的一年。纵观国际国内形势，机遇和挑战并存，希望与困难同在。我们要珍惜来之不易的发展势头；珍惜行之有效的发展思路；珍惜国家“扩内需、稳增长、惠民生、调结构”的总体要求；珍惜国家继续实施积极的财政政策和适度宽松的货币政策、保持经济平稳较快发展的一揽子计划；珍惜亿吨煤电基地、皖江城市带承接产业转移示范区、合肥经济圈建设、城乡一体化综合配套改革试点等带来的难得机遇；珍惜我市人均GDP跨过3000美元、工业化和城镇化快速推进中蕴藏的巨大需求；珍惜丰富的资源优势、较强的产业基础、深厚的文化底蕴、相对优越的区位交通等支撑条件；珍惜全市上下推进科学发展、加速淮南崛起的创业激情和团结拼搏、奋发有为的精神风貌。这是我们应对挑战、奋力赶超的强大动力。我们要增强忧患意识，坚定必胜信心，认清形势、看清走势、把握趋势，迎难而上、知难而进、克难攻坚，为夺取应对金融危机的全面胜利，为“十二五”规划的启动实施，奠定更加坚实的基础。
做好今年的政府工作，要以邓小平理论和“三个代表”重要思想为指导，深入贯彻落实科学发展观，紧紧围绕资源节约型和环境友好型的“两型城市”建设，立足煤、延伸煤、不惟煤、超越煤，加快以工业化和城市化双轮驱动为主战略、以合淮同城化和城乡一体化为侧翼的“一主两翼”发展，着力推进创新转变发展方式，着力调整结构提升产业层次，着力改善民生扩大消费需求，着力改革开放增强发展活力，着力统筹协调促进社会和谐，努力保持经济社会平稳较快发展的良好势头，在新的起点上推动科学发展、促进城市转型、加速淮南崛起。

      今年要在做好“十一五”收官的同时，把谋划“十二五”与建设“两型城市”紧密结合起来，按照“全面规划、项目支撑、政府主导、市场运作、深化改革、有效推进”的原则，积极探索有利于资源节约和环境保护的体制机制，促进可持续发展；探索有利于增强自主创新能力的体制机制，推动创新型城市建设；探索有利于加快先进制造业和现代服务业发展的体制机制，优化产业结构；探索有利于集约节约用地的体制机制，走集约型城市化发展道路；探索有利于解决城乡二元结构的体制机制，推进城乡一体化发展；探索有利于合淮同城化的体制机制，促进区域经济协调发展，努力把淮南打造成为综合实力较强、经济结构优化、集聚功能较好、创新活力迸发、可持续发展的资源节约型和环境友好型城市。

      今年经济社会发展的主要预期目标是：地区生产总值增长12％，财政收入增长14％，固定资产投资增长30％以上，社会消费品零售总额增长16％，城镇居民人均可支配收入增长8％，农民人均纯收入增长10％，新增城镇就业岗位2.7万个，城镇登记失业率控制在4.5％以下，人口自然增长率控制在7.5‰以内，单位生产总值能耗下降4.3％，二氧化硫排放量基本持平，化学需氧量削减2.2％。

      为实现全年目标，着力抓好五个方面工作。

**（一）千方百计力推工业化，提高经济发展质量和效益**

      按照产城一体、“T”型布局、扬煤凸企、扬长补短的基本思路，一手抓深煤、一手抓非煤，一手抓新兴产业培育、一手抓传统产业改造，推动结构调整和产业升级。

      全年完成工业投资140亿元，新增储备项目100个以上，新增规模以上工业企业130户以上，工业增加值增长16%以上。
打造“T”型布局，引领工业化。坚定不移地推进“T”型空间布局，继续完善以沿淮产业带、合淮工业走廊为主线的规划布局和功能，提高承载力和吸引力。坚定不移地推进“T”型产业布局，山南重点建高新区和低碳区，山北至淮河以南重点建加工区和服务区，淮河以北重点建重化工业区。

      加快园区建设，承载工业化。坚持把园区作为工业发展的有效平台和结构调整的重要抓手。强力实施市经济开发区“倍增计划”，全年引进内资、利用外资、完成投资、新增规模以上工业企业增长一倍以上，分别达到20亿元、1000万美元、20亿元、22户。支持市经济开发区申报国家级开发区。加快农垦共建区建设，抓好东部工业园、农场危旧房改造、安徽德普胶带等一批项目开工，确保力达电气、高档粘胶纱等一批项目竣工。加快申报淮南高新技术产业开发区为省级高新技术开发区，完成启动区一期路网工程建设，引进一批科技创新型企业进驻高新区，促进电子八所3G光纤光缆组件等一批项目落户高新区。加快煤化工基地、潘集工业集聚区申报省级开发区和高新技术产业基地。推进淮南工业园、凤台开发区建设。支持毛集工业集聚区申报省级开发区，支持大通工业园、安成工业集聚区和八凤工业走廊加快发展。启动合淮共建区建设。抓好凯盛重工、淮河化工等16户企业退城进园工作。

      培育支柱产业，加速工业化。编制百亿产业发展规划。推进煤电规模化经营、集约化利用、现代化装备、安全化生产。加快朱集西矿、朱集东矿、杨村矿、平电三期、淮沪二期、淮浙二期、1000千伏特高压等项目的核准和开工。实施煤化工产业大区域布局、大项目策划、大集团引领、大基地承载。促成中石化合资公司在淮尽快注册，确保170万吨甲醇转化制烯烃项目上半年开工。抓好110千伏变电所、30万吨自来水厂、10万吨污水处理厂、热电联供等配套基础设施建设。加快淮化27万吨浓硝酸、德邦100万吨纯碱二期建设。积极发展高效载能工业，加快煤-电-铝、煤-电-硅-光伏一体化发展。大力发展循环经济，支持淮南矿业、鑫旺建材等省级循环经济试点企业发展，抓好顾桥煤矸石电厂等项目开工，不断提高煤矸石、粉煤灰的综合利用水平。以成套化、专业化、高端化为方向，加强与郑州煤机合作，促成比塞洛斯煤机、长壁煤机建成投产，促进省级煤机装备制造基地上规模、上水平。结合产业调整振兴规划，实施一批重点项目，推动电子信息、生物医药、新材料、轻工、纺织等产业发展壮大。用3年左右的时间，培育百亿产业4个。

      培育大企业，支撑工业化。组织编制亿元企业、百亿元企业发展规划，实行规模以上工业企业排名制度。支持淮南矿业、国投新集、皖北煤电等大型企业发展，不断壮大总部经济。全年新增销售收入超亿元的企业5户以上、超5000万元的企业10户以上。用3年左右的时间，培育销售收入超500亿元的企业1户、超100亿元的2户、超50亿元的3户、超亿元的100户。

      培育名牌产品，夯实工业化。积极发展精深加工产品、高附加值产品，大力培育拳头产品、特色产品、名牌产品，新创省名牌产品4个、省著名商标15个，力争实现中国驰名商标零的突破。坚持质量第一，全面推进“市长质量奖”工作，鼓励企业提高产品质量，提升市场竞争力。

      加快以民营经济为重点的非公经济发展，促进工业化。实施民营经济发展“腾飞”计划，营造鼓励和支持非公经济发展的良好环境。制定民营经济产业指导目录、重点民营企业发展名录。建成中小企业服务中心2家。选择50家民营企业予以重点支持，确定50家民营企业实行挂牌保护。全年新增民营企业1000户以上、个体工商户3500户以上，非公经济增加值增长20%以上。
推进科技创新和节能减排，提升工业化。推动培育战略性新兴产业与核心技术、优势产业相结合，用高新技术和先进实用技术改造传统产业，提升产业档次。实施500万元以上技术改造项目80个。围绕煤化工、瓦斯治理、生物医药等重点领域，激发高等院校和科研院所科技创新活力，加强技术攻关。加快煤矿瓦斯治理国家工程研究中心、生态环境保护国家工程实验室、国家煤及煤化工产品质检中心、淮南科技创新公共服务中心建设，建成3家省级企业技术中心，新建5家省、市级工程技术研究中心。大力引进科技创新型人才，构建有利于嫁接、共享和开放的创新体系。全年认定高新技术企业8家，申报省级技术创新项目60项，开发新产品100个，申请专利1000件。完成高灰熔点淮南煤气化工业技术试验。支持毛集国家可持续发展先进示范区建设。积极开展低碳经济试点，着力发展低碳产业，充分发挥碳汇减排功能。引导人们转变生活方式、消费方式，推动经济社会向低碳社会转型。抓好27户重点企业节能降耗，加快淘汰落后产能，全面完成“十一五”节能减排任务。

      强化政策措施，服务工业化。着力启动“工业结构调整年”，健全加快工业改革发展的政策体系。出台加快推进工业化的实施意见，完善结构调整和产业升级、鼓励工业项目投资、非公经济发展、国有资产保值增值的政策措施。建立健全工业化的推进机制，强化组织保障，完善资金支持、项目推进、运行监测、技术进步和目标考核机制。统筹安排工业发展支持资金8700万元，加大专项支持、科技创新和上市培育力度。出台加快装备制造业和医药产业发展实施方案。

**（二）大力推进城市化，创造良好的城市面貌和人文环境**

      围绕打造“山水园林城市、滨河滨湖城市、宜居宜游宜学宜业”城市，坚持科学规划引领，树立精品精致标准，采取市场运作模式，以人民满意为目标，不断完善城市综合服务功能，提升城市综合承载能力，努力打造独具魅力的淮河明珠。

      全年完成大建设投资200亿元以上，确保力度不减、势头不降、劲头不松。

      以大规划引领城市大建设。启动滨河新城、滨高塘湖新城规划和建设，编制滨瓦埠湖新城概念性规划、八公山与卧龙山一体化规划。完成西部沉陷区生态治理规划和医疗卫生设施、停车场等专项规划编制。加快主城区20平方公里控制性详细规划、洛河洼和蔡城塘周边概念性规划编制，抓好高铁站周边地块设计。完成八公山综合旅游服务区规划、职教园区控制性详细规划、山南水系补水规划编制和20平方公里地形图补测补绘、建成区地形图航测。一是新区开发有新规模。推进南纬一路二期、南经十六路、水系景观改造等7个基础设施项目建设，加快淮南大剧院（人民会堂）、市科技馆、市委党校和公交站场等一批社会事业项目建设，开工建设职教园区、安徽理工大学新校区、少儿艺术研发基地、市妇幼保健院、华仑国际文化广场等一批社会投资项目，确保市级机关办公大楼、武警机关新营区等项目竣工。实施重点商业住宅开发项目10个以上，新开工面积200万平方米，总投资50亿元以上。积极引进大型城市综合体入驻山南。规划建设山南会展中心。二是老区提升有新形象。抓好石姚湾退建区的综合利用和开发，还道于水的5平方公里集中发展短期作物，保留的15平方公里集中进行城市开发和工业布局。实施龙湖公园周边等危旧房改造，整治旧小区40个。开工建设朝阳东路等12个延伸工程、人民北路等10个改扩建工程、广场路等4个下穿立交工程和2个过街人行通道，加快淮舜南路等20条道路建设。结合老城道路网改造，推动城市道路交通安全项目建设，优化道路交通安全系统，实施公交优先战略，提升人行道标准。规划并实施国庆路、舜耕路等道路亮化工程，新改建16条道路路灯。启动大别山饮用水源工程，抓好西部垃圾处置设施建设。开工建设7个城市防洪排涝工程。三是矿区改造有新面貌。加大棚户区改造和矿区修复力度，推进32个棚户区改造项目建设，新开工面积210万平方米。加快泉大资源枯竭矿区环境修复与开发。四是景区完善有新档次。开工建设淮南志高文化科技动漫产业园，积极引进美国侏罗纪建设舜耕山主题公园，打造舜耕山景区。规划建设大通老矿区生态体育文化广场。推进八公山、卧龙山和茅仙洞一体化开发。推进上窑山、上窑镇和高塘湖一体化建设，抓好高塘湖中心岛休闲渔业开发。抓好龙湖公园、茅仙洞争创国家4A级风景区工作。加快大通万人坑爱国主义教育基地建设。启动淮河公园、淮西湖公园建设，续建十涧湖国家城市湿地公园。建成10个省优秀旅游乡镇。五是立体交通有新提升。加快淮蚌高速公路建设，推进淮滁高速公路前期工作，确保206国道改线工程、东西部第二通道等建成通车。积极争取商杭高铁经过淮南并设站，加快合淮蚌高铁暨淮南东站建设。加快孔李淮河大桥建设，开工建设淮河二桥，争取凤台二桥开工。大力发展淮河水运，完成淮南新港、煤化工基地专用码头、海螺专用码头等一批项目前期工作。六是承办省运会有新水平。瞄准省内一流的目标，高标准建设全民健身综合训练馆、市水上运动训练基地等场馆。建设40个农民体育健身工程。

      以大建设推动服务业大发展。统筹发展生产性服务业和生活性服务业，改造提升传统服务业，加快发展现代服务业。出台支持服务业发展的政策措施。加强服务业统计和项目库建设。突出抓好五个方面：一是着力提升商贸物流业。启动新老城区大型城市综合体前期工作，加快东方国际购物中心和产业街、特色街建设，打造龙湖路商圈。进一步壮大淮矿物流园，加快安成物流园、医药物流园和曹庵工业物流园建设，建成鑫海物流园，规划建设东部物流园。二是加快发展房地产业。统筹经济适用房和廉租房建设、棚户区改造、沉陷区村庄搬迁、商住开发，全年房地产新开工面积410万平方米以上，竣工200万平方米以上。加强房地产市场规范运行。三是大力推进文化产业和文化事业发展。坚持以文化软实力提升经济硬实力，培育电影、动漫、演艺等文化产业，制作发行动漫系列《淮南子传奇》，开拍以花鼓灯为题材的电视剧《拯救》、以乡村为题材的电视剧《承诺》。加快家乐福横店影视城、西部书城、海顿影视基地等一批项目建设。积极筹建新图书馆和新艺术馆，建成乡镇综合文化站39个、农家书屋100个。四是加快发展金融服务业。争取引进股份制商业银行2家，组建村镇银行1家。抓好小额贷款公司开业和规范运行，积极争取新获批5家。加强银企对接，开办“金融超市”。成立担保行业协会。建立中小企业信用库。支持担保商会发展。五是积极培育信息等服务业。推进服务产品和方式创新，加快电子政务和电子商务发展。大力发展会展、法律、会计等服务业。

      以大创建带动城市形象大提升。争创第二届全省创建文明城市工作先进市。进一步完善“两级政府、三级管理、四级网络”的城市管理体制，建立城管、公安、法院协同执法机制，形成“大城管”格局。进一步明确市与区、条与块的职责分工，加强数字化指挥、网格化管理、精量化定责、精准化操作。强力拆除和制止违章违规违法建设。规范中巴车、出租车、户外广告管理。强化市民交通意识、环保意识和卫生意识，大力提高市民文明素质，共同建设我们的美好家园。全面推进全国绿化模范城市创建。着力构建“三山鼎立”的绿色屏障体系，“三水环绕”的绿色河湖体系、“三城互动”的绿色景观体系、“三矿并举”的绿色矿区体系、“三农共建”的绿色乡村体系、“三路协调”的绿色长廊体系。成片造林1.3万亩，新增城市绿地100万平方米。进一步开展“净化社区、美化街巷”活动。办好第十二届全省运动会、第十七届中国豆腐文化节和第十届全国“市长杯”乒乓球赛，事关淮南形象，倍受省内外瞩目，要以“五彩淮南”的崭新形象，迎接精彩的节会。

**（三）持之以恒推进城乡一体化，完善统筹发展的体制和机制**

      全面实施省城乡一体化综合配套改革试点市工作，核心是推进“三集中”，重点是发展非公和县区域经济，目的是破除城乡二元结构。加快制定城乡一体化综合配套改革实施方案，抓好城乡一体化发展总体规划、各专项规划的编制和实施。积极申报省和国家现代农业示范区。

      全年编制农业项目100个以上，农业项目库储备规模达50亿元，重点加快60个在建项目的实施。

      大力推进城镇化，拓展内需增长空间。紧紧围绕市矿统筹、城乡规划统筹、产业发展统筹、社会建设统筹、扩权强镇和农村综合配套改革统筹，突出抓好三件事。一是加快企业向园区集中。按照“布局集中、用地集约、产业集聚”的原则，完善支持县区工业园区发展的政策措施，推动园区产业关联发展、成链发展、集约发展、合作发展。支持省扩权强镇试点镇规划建设工业集聚区，着力培育4个省级专业集群镇。二是加快人口向城镇集中。推进户籍制度改革，放宽城镇户籍限制。深入实施“千村百镇”和“1860”示范工程。加快上窑洛河一体化、舜耕安成滨河型城镇建设和山南七乡镇发展。把推进城镇化与采煤沉陷区综合治理结合起来，科学制定采煤沉陷区村庄搬迁安置计划，编制综合治理、土地整治规划及项目库。加快采煤沉陷区省试点工程实施，支持凤台环城北湖新区建设，推进大集中安置。推广采煤沉陷区治理“后湖”模式，改善涉矿乡镇生产生活环境。三是加快土地向规模集中。加强土地整理和置换，实施整村推进，整理土地1万亩，置换土地0.35万亩。大力推进农村土地承包经营权流转改革，流转面积12万亩以上，鼓励发展适度规模经营。积极借鉴“两分两换”的土地流转模式，逐步实现“土地节约集约有增量、农民安居乐业有保障”的目标。

      大力发展现代农业，拓宽农民增收渠道。贯彻落实强农惠农政策，稳步提高粮食收购价格，扎实开展涉农资金整合，全面提升农业综合生产能力。全市财政投入支农资金16亿元，增长14.3%。突出优质化，加强百万亩优质小麦、优质水稻示范区建设，实现百万吨优质粮食目标。突出特色化，加快特色蔬菜、特色经果林、特色养殖基地发展，规划建设农业科技示范园区。突出产业化，加快益益乳制品加工基地建设，建成八公山豆腐文化产业园，开工建设农产品加工园区，争创省级以上农业产业化龙头企业3家、市级40家，农产品加工业实现产值100亿元，增长23%以上。突出规模化，大力发展“公司+农户”、订单农业等生产模式，支持种植大户、养殖大户扩大生产规模，促进产供销一条龙、贸工农一体化发展。突出标准化，争创无公害农产品、绿色食品、有机食品15个以上，建成农业标准化示范区10个以上。完善农村综合服务体系，整合农村专业合作组织30个，建成高标准农技推广站10个，深入推进“万村千乡”市场工程、“新网工程”，继续开展家电、汽车摩托车下乡活动。培训新型农民1.8万人。支持民族乡发展。完成10个村扶贫开发整村推进任务。

      大力推进农村基础设施建设，拓展公共服务领域。一是推进治淮工程建设。完成石姚段、洛河洼行洪堤退建加固工程，争取开工西淝河、高塘湖洼地治理应急工程和焦岗湖、港河、架河平原洼地治理项目，做好董峰湖行洪区调整改造前期工作，加快上、下六坊堤废弃和汤渔湖行洪区调整改造项目立项。实施朱集灌区节水改造、禹王大型泵站更新改造和泥河泵站应急维修，完成36项农田水利基本建设。二是推进农村饮水工程建设。解决11.65万农村人口安全饮水问题。三是加强农村公路养护。落实农村公路养护政策措施，建立健全长效养护机制。新建农村客运站7个。四是推进农村环境整治。继续实施“清洁乡村、美化家园”示范工程，新增试点村100个以上。新建5个村镇生态式污水处理站，加快创建省级环境优美乡镇步伐。五是实施退网还湖工程。坚持退网还湖与滩涂养殖规划、渔民生活保障、旅游业发展、城镇化推进、滨湖城市打造相结合，加快焦岗湖、高塘湖、瓦埠湖退网还湖，新增退网还湖面积1.5万亩。

**（四）坚定不移推进合淮同城化，拓展改革开放广度和深度**

      扩大开放胸怀，创新开放政策，优化开放环境，统筹外宣、外资、外经贸、外智、外事工作，在更宽领域、更深层次、更大范围推进开放、促进发展。

      加速合淮同城化。落实与合肥、六安既定的合作框架协议，提升淮南在合肥经济圈的作用。开工建设淮南大道、九龙大道，做好舜耕大道、淮南至新桥机场快速连接线前期工作，启动城市快速公交系统、城际轻轨、新桥机场淮南航站楼规划，参与引江济淮工程规划。按照合肥经济圈城镇体系规划中的功能定位，打造以煤电化产业为主导的国家亿吨煤基地、华东火电基地、安徽省重化工基地、合肥经济圈带动沿淮、辐射皖北的中心城市及门户。借助皖江城市带承接产业转移示范区平台，发挥综合优势，创新援建、托管、股份合作等共建模式，推动与发达地区合作建设共建区，加快“飞地”经济发展，努力打造面向长三角的产业承接基地、能源原材料基地、劳务输出基地、旅游休闲基地。

      加大招商引资力度。结合产业、资金、人才流动的新趋向，瞄准重点区域，盯准意向企业，找准招商方式，努力实现招商引资新突破。全年利用外资增长12%以上，引进内资增长30%以上。大力推行小分队招商、专业化招商和节庆招商等方式，组建深圳、厦门、杭州招商联络处，实行“一把手”定期带队招商，大力开展境内外招商推介，积极组织参加中博会、徽商大会等节会。加强对招商引资的目标考核、奖惩激励，以硬约束倒逼硬指标，完成硬任务。积极培育进出口孵化体系，实现进出口总额增长10%。建立健全与央企合作互动机制，加强25个签约项目协调、调度与推进，逐一落实开工条件。继续策划包装一批大项目、好项目，寻求与央企省企更大的合作。

      加强投融资平台建设。发挥各类融资平台功能，推进城投、产投公司市场化改革，分别实现融资50亿元、30亿元。做大山南和垦区投资公司，筹建文化、旅游、林业和各县区投资公司，积极探索城投、产投与其它公司的母子公司体制关系。统筹安排财政资金68亿元，撬动民资，吸引外资。用足、用活、用好扩大内需政策，积极争取中央新增投资、省级配套资金和境外金融机构贷款。

      加快各项改革。推进淮南矿业、淮化集团上市工作，加快4户中小企业上市。完成皖淮机械厂、汽运总公司改革重组，实行淮化集团主辅分离，争取皖纸公司资产重组。妥善处置“出中心”国有企业资产，整合企业留守处。基本完成不良金融资产打包处置工作。强化国资监管，确保国有资产保值增值。完成政府机构改革，理顺部门职能关系。积极推进事业单位改革。加快文化体制改革。继续深化农村改革。加快9个省级扩权强镇试点镇的改革。做好农村集体土地所有权确权登记颁证工作，推行城乡建设用地增减挂钩。稳妥推进价格改革。

**（五）坚持不懈改善和保障民生，全力维护社会和谐稳定**

      坚持新增财力向困难群众倾斜、向农村倾斜、向基层倾斜、向社会事业倾斜，逐步实现“学有所教、老有所养、病有所医、劳有所得、住有所居”的目标。进一步加大投入、巩固成果、扩大范围、提升标准、健全机制，重点实施42项民生工程。

      坚持教育优先发展、均衡发展和规范发展。出台加快义务教育均衡发展若干意见。市本级教育支出预算安排增长17%。推进中小学布局调整和标准化学校建设。大力实施校舍安全工程，改造校舍90万平方米。加快发展高中教育，促进职业教育资源整合和布局调整。重视学前教育，关心特殊教育，鼓励民办教育，提升高等教育。

      建立健全公共卫生服务体系。一是完善医疗服务体系。规划建设江淮心脑血管病中心医院，建成市医疗废物处置中心、马山医院无障碍住院病房、100所标准化村卫生室和16个社区卫生服务机构。二是完善医疗保障体系。提高新型农村合作医疗补助补偿标准，全面推行门诊统筹，实现“即看即报”；城镇居民和城镇职工基本医疗保险实现应保尽保。三是完善公共卫生服务体系。完成11万重点人群甲型H1N1流感疫苗接种，新建2万座无害化厕所，加强重大传染病防控和重点人群健康服务，全面推行儿童免费预防接种。四是建立健全药品供应体系。严格实施国家基本药物制度。五是抓好凤台县、田家庵区基层医药卫生体制综合改革试点。六是稳定现行生育政策和低生育水平，加强计划生育县站乡所规范化建设。做好第六次人口普查工作。

      大力促进就业再就业。坚持以项目扩大就业，以园区吸纳就业，以创业带动就业，以培训促进就业。充分发挥创业园、实训基地和劳动保障服务平台作用，深入开展采煤沉陷区农民培训就业援助行动，统筹高校毕业生、失地农民、返乡农民工、失业人员、复转军人就业。新增下岗失业人员再就业1.7万人、转移农村劳动力3.2万人，创业就业培训3.65万人。

      完善社会保障体系。围绕缴费标准最低化、报销比例最大化、服务举措最优化，完善“五保合一”、“一票多费”征缴制度。推进凤台县新型农村社会养老保险试点，加强养老保险政策整合，逐步扩大养老保险政策覆盖范围。扩大失业金支出范围，规范工伤管理，优化生育保险结算程序。加强对失地农民就业服务和社会保障。逐步完善社会救助体系，探索建立城乡医疗救助“一站式”服务。适时调整最低生活保障标准，缩小城乡差距。新建、改扩建农村敬老院10所，五保集中供养率提高到38%。建立残疾人综合服务体系。加快发展社会福利、慈善和老龄事业，开工建设市社会福利院综合楼、市流浪未成年人救助中心、市光荣院。采取市场化方式，筹建市级老年公寓。加快廉租房和经济适用房建设。

      推进安全发展。加强社会治安综合治理，推进公安“三项建设”，依法打击违法犯罪活动，完善综合防控体系。积极开展信访大排查、大调处、大接访、大下访、大化解，推动关口前移、重心下移，最大限度地化解积案、减少新案，努力把矛盾化解在基层。坚持预防与应急并重、常态与非常态结合，提升防灾救灾水平。加强应急平台建设，推进应急管理进社区、进农村、进企业、进学校。严格落实安全生产责任制和责任追究制，构筑群防、群控、群治的安全生产网络，突出煤矿、非煤矿山、食品药品等重点领域安全监管，支持国投新集办好第八届全国矿山救援技术竞赛活动，坚决防范和遏制重特大安全事故，切实为人民群众撑起安全的“保护伞”。

      加强民主法治建设。自觉接受人大法律监督和政协民主监督，认真办理人大代表议案、建议和政协委员提案，充分听取各民主党派、工商联、无党派人士及各人民团体的意见，广泛接受社会公众和新闻舆论监督。加强和改进政府法制工作。提请市人大常委会出台交通安全管理等3个条例，修改市采煤塌陷地治理、城市绿化、市政工程设施管理3个条例，草拟城市拆迁管理条例及制止城乡违法违规建筑条例。积极支持驻淮部队建设，做好国防动员、民兵预备役和人防工作，提升双拥优抚安置工作水平，巩固发展军政军民团结。

**三、奋发有为，着力形成齐头并进的合力**

      特殊时期，特殊考验，对政府工作提出了特殊要求。政府工作人员特别是领导干部要有特殊精神，做出特殊努力，切实提高施政水平，更好地为改革发展大局服务、为全市人民群众服务。

      要强化以谋划发展为龙头的能力建设。牢固树立超前谋划的大局意识、忧患意识，善于从形势变化中把握发展机遇，善于从深入学习中理清发展思路，善于从赶超实践中创新发展方式,善于从群策群力中破解发展难题，抓好全局性、牵动性、导向性的大事。坚持统筹兼顾，有效整合行政资源、自然资源、资本资源、人力资源、信息资源，协调好局部与整体、当前与长远、改革发展稳定的关系，着力提高解决实际问题的能力、化解复杂矛盾的能力、促进科学发展的能力。

      要强化以实干实效为核心的务实作风。加快淮南崛起，关键在实干。完成今年任务，关键在落实。要弘扬“五加二”、“白加黑”的实干精神、争分夺秒的抢先精神、迎难而上的攻坚精神、敢想敢干的拼搏精神、埋头苦干的务实精神，用超常的办法、超常的干劲做工作、抓落实、求实效。大幅精简会议、文件，大力改进会风、文风。坚持加快发展不动摇、不折腾、不懈怠，向市场要项目、要资金，用一流的标准抓项目、抓投资，努力在新一轮发展中抢占先机、赢得先发。

      要强化以机制创新为抓手的闭环管理。坚持决策、执行、监督、反馈、考评等环节有机统一，逐步在政府、部门和行业之间形成闭环。进一步推进和完善科学民主决策机制，坚持问政于民、问需于民、问计于民，深入调查研究，广泛听取意见。对重大决策和部署，要明责任、抓到位、见效果，严格执行纪律，确保政令畅通、令行禁止。落实“五位一体”大督查机制，全面提升政府执行力。继续清理行政审批事项，加大向县区放权力度。巩固“两集中、两到位”成果，完善三级联动服务体系，创新审批流程，推行网上办事，实行“一窗式”全程代理服务，落实“只进一个门、只找一个人”承诺。加强公务员队伍建设，认真开展考核评议工作。依法推进政务公开，更加注重公共权力大、公益性强、公众关注度高的部门和领域的信息公开，坚决防止行政不作为和乱作为。充分保障人民群众的知情权、参与权、表达权和监督权。

      要强化以廉洁清正为基石的政府形象。贯彻“为民、务实、清廉”的要求，建立健全惩防体系，认真落实“一岗双责”。加强和改进政风行风评议，切实纠正损害群众利益的不正之风。强化对政府关键岗位、重点领域、重要环节的监督制约，严防权力寻租。完善政府投资监管，规范工程招投标、土地使用权出让、政府采购和产权交易工作。加大专项治理力度，对违法违纪行为，决不姑息，决不迁就，决不手软。深化公务消费和公务接待制度改革，树立廉洁勤政的良好形象。

      各位代表，我们面临的任务艰巨而繁重，我们肩负的责任重大而光荣。让我们紧密团结在以胡锦涛同志为总书记的党中央周围，高举中国特色社会主义伟大旗帜，深入贯彻落实科学发展观，在市委的坚强领导下，增强逆水行舟的紧迫感，鼓足知难而进的精气神，扎实工作，锐意进取，为推进淮南跨越发展、夺取全面建设小康社会的新胜利而努力奋斗！
